# Personal

**Published:** 1912-02

**Authors:** 


### PERSONAL

Our readers are requested to send items for this section.

Dr. M. A. King of Rochester, has moved from 80 Clinton Avenue, South to 21 East Avenue. Office hours, 1 to 3 and 7 to 8.

Dr. Douglas P. Arnold of Buffalo, has been appointed Medical School Inspector.

Dr. Regina Flood Keyes and Dr. Mabel Flood of Buffalo, sailed late in January for Cuba, Jamaica and Panama.

Dr. Fritz Reichmann, State Superintendent of Weights and Measures, declares Troy to be the worst short weight city in the state. Well, one shouldn't expect Avoirdupois weights in Troy.

Dr. L. J. Knell of Buffalo, was elected President of the New York State Council of Elementary School Principals and Teachers, at Syracuse, December 30, 1911. Dr. Knell is a graduate of the Medical Department of the University of Buffalo, who has done his part in the most practical way possible, toward solving the problem of an overcrowded medical profession.

Dr. Henry B. Lyon is mayor of Dunkirk. Sir Thomas Boor Crosby, M. D. is the 723d Lord Mayor of London, and the first physician thus honored. He is 81 years old, and is an ex-president of the Hunterian Society. Dr. Sun, is President of China. Doctors ought to make good executives for their whole training is to force results under difficulties. Still, there is an old motto: "Ne sutor ultra crepidam."

---

Dr. Theresa Bannan of Syracuse, has recently published a valuable historic and genealogic work on the Irish of Onondaga, County.

---

The editor acknowledges the hospitality of Dr. Fayette H. Peck, of Utica, retiring President of the Oneida County Society, and the cordial reception of the members, during a recent trip to Utica.

---

Dr. Robert J. Talbot has been appointed Health Officer of Niagara Falls.

---

Dr. E. E. Gillick of Niagara has been appointed a member of the Board of Education.

---

Dr. J. F. Crowley of Batavia has been appointed physician for the town poor.

---

Dr. John M. Swan has resigned as Medical Superintendent of the Glen Springs, of Watkins.

---

Dr. F. Parke Lewis of Buffalo has returned from Europe.

---

Dr. G. F. Smith of Falconer, gave a lecture on the Relation of Alcohol to Health, before the T. P. C. T. U., January 26, 1912.

---

Dr. H. M. Edmunds was appointed Health Officer of Tonawanda, January 26, 1912.

---

Dr. C. R. Brown, has returned to Perry, after some time spent in Buffalo.

---

D. H. Squier, D. D. S. of Buffalo, was elected Vice-President of the Institute of Dental Pedagogics at Chicago, January 27, 1912.

---

Dr. C. W. Clendenan of North Tonawanda sustained a fracture of the fore-arm while cranking his automobile, January 28, 1912.

Dr. C. S. Jones of Pittsburgh, was the guest of Dr. A. E. Hubbard of Buffalo, late in January.

---

Dr. A. Vander Veer of Albany writes us from the Hotel Clarendon, Sea Breeze, Fla.

---

Dr. Charles Cary of Buffalo is in Panama.

---

Dr. Charles D. Aaron of Detroit delivered the opening address at the beginning of the 61st session of Heidelberg University, Tiffin, Ohio.

---

During a recent professional trip to Lockport, we had a pleasant visit with one of our oldest subscribers, Dr. C. N. Palmer. Dr. Palmer is the grandson of a Revolutionary soldier and has, in his possession, the gun carried by the latter before he became an officer. It may be of interest to note that the last Revolutionary veteran died about the time that the Civil War began. It is doubtful whether any Sons and Doughters, in the literal sense survive, though several lived well into the present century, and even grandchildren are few.

---

Dr. Clifford V. C. Comfort of Rochester (University of Buffalo, 1897), has been nominated for a lieutenancy in the Medical Corps of the National Guard.

---

Dr. Myron B. Palmer of Rochester, has been elected president of the Hospital Medical Society.

---

Dr. J. W. Magill (Buffalo 1887) and Dr. O. E. Jones, have been appointed Surgeons to the Rochester General Hospital.

---

Dr. William F. Frasch of Rochester (University of Buffalo 1902), is making a good recovery following an operation for strangulated hernia.

---

Dr. Henry T. Williams of Rochester, has been appointed on the Park Board.

